# Fabrication of *β*-Ga_2_O_3_ Nanotubes via Sacrificial GaSb-Nanowire Templates

**DOI:** 10.3390/nano13202756

**Published:** 2023-10-13

**Authors:** Lei Shangguan, Long-Bing He, Sheng-Pan Dong, Yu-Tian Gao, Qian Sun, Jiong-Hao Zhu, Hua Hong, Chao Zhu, Zai-Xing Yang, Li-Tao Sun

**Affiliations:** 1 SEU-FEI Nano-Pico Center, Key Laboratory of MEMS of Ministry of Education, Southeast University, Nanjing 210096, China; 230198521@seu.edu.cn (L.S.); 220221733@seu.edu.cn (Y.-T.G.); 220221749@seu.edu.cn (Q.S.); huahong@seu.edu.cn (H.H.); phczhu@seu.edu.cn (C.Z.); slt@seu.edu.cn (L.-T.S.); 2 SEU-AMTE Collaborative Center for Atomic Layer Deposition and Etching, Southeast University, Wuxi 214000, China; 19906783878@163.com (S.-P.D.); 220226133@seu.edu.cn (J.-H.Z.); 3 School of Physics, Shandong University, Jinan 250100, China; zaixyang@sdu.edu.cn; 4 School of Microelectronics, Shandong University, Jinan 250100, China

**Keywords:** *β*-Ga_2_O_3_, nanotube, nanowire template, Kirkendall effect, electrical property

## Abstract

*β*-Ga_2_O_3_ nanostructures are attractive wide-band-gap semiconductor materials as they exhibit promising photoelectric properties and potential applications. Despite the extensive efforts on *β*-Ga_2_O_3_ nanowires, investigations into *β*-Ga_2_O_3_ nanotubes are rare since the tubular structures are hard to synthesize. In this paper, we report a facile method for fabricating *β*-Ga_2_O_3_ nanotubes using pre-synthesized GaSb nanowires as sacrificial templates. Through a two-step heating-treatment strategy, the GaSb nanowires are partially oxidized to form *β*-Ga_2_O_3_ shells, and then, the residual inner parts are removed subsequently in vacuum conditions, yielding delicate hollow *β*-Ga_2_O_3_ nanotubes. The length, diameter, and thickness of the nanotubes can be customized by using different GaSb nanowires and heating parameters. In situ transmission electron microscopic heating experiments are performed to reveal the transformation dynamics of the *β*-Ga_2_O_3_ nanotubes, while the Kirkendall effect and the sublimation process are found to be critical. Moreover, photoelectric tests are carried out on the obtained *β*-Ga_2_O_3_ nanotubes. A photoresponsivity of ~25.9 A/W and a detectivity of ~5.6 × 10^11^ Jones have been achieved with a single-*β*-Ga_2_O_3_-nanotube device under an excitation wavelength of 254 nm.

## 1. Introduction

Ga_2_O_3_ nanostructures have attracted intense interest because of their promising applications as functional devices [1,2,3,4,5,6,7,8,9,10,11]. Among them, one-dimensional Ga_2_O_3_ nanocrystals including nanowires, nanobelts, nanorods, and nanotubes are demonstrated to be the ideal materials for high-performance electronic devices [12,13,14], sensors [15,16,17], solar-blind photodetectors [16,18,19,20], and catalyzers [21,22], benefiting from their high surface-to-volume ratios. For example, Delaunay et al. achieved a solar-blind photodetector based on the *β*-Ga_2_O_3_ nanowires [5], and the device possesses a very fast decay time (<20 ms) and a quite-low photocurrent fluctuation (<3%). The 250-to-280 nm rejection ratio of this device is as high as ~2 × 10^3^, exceeding that of the devices fabricated with bulk *β*-Ga_2_O_3_ single crystals (with a 250-to-280 nm rejection ratio of ~20). Hu et al. achieved a photodetector using *β*-Ga_2_O_3_ nanobelts [20]. The photodetector has a high photo-excited current of over 21 nA (with a dark current below 10^−14^ A), a high responsivity of around 851 A/W, and a high external quantum efficiency of around 4.2 × 10^3^. These performances are superior to the photodetectors using materials such as In_2_Ge_2_O_7_ and Zn_2_GeO_4_. Lu et al. fabricated a field-effect transistor based on Nb-doped *β*-Ga_2_O_3_ nanobelts [13]. The device shows excellent electrical performances such as an ultra-small cut-off current of around 10 fA and a high on–off ratio over 10^8^. These pioneering devices suggest that the *β*-Ga_2_O_3_ nanostructures/arrays can provide more energetic surfaces and activated sites, which thereby promote the device performances in comparison with their bulk counterparts.

Aside from nanowires and nanobelts, *β*-Ga_2_O_3_ nanotubes are another sort of one-dimensional nanostructure which possess much higher surface-to-volume ratios. Their hollow structures have intrinsic advantages as they provide an extra degree of freedom to adjust the structure and property. Unfortunately, the development of *β*-Ga_2_O_3_ nanotubes is severely hindered as the fabrication of tubular nanostructures is still challenging. Unlike the *β*-Ga_2_O_3_ nanowires/nanobelts for which the fabrication methods such as chemical vapor deposition [23,24,25,26,27,28], arc discharge [29], solution synthesis [30], and laser ablation [31] have been proven to be feasible, the fabrication methods for *β*-Ga_2_O_3_ nanotubes are rare, while tubular structures of *β*-Ga_2_O_3_ are seldom reported. Although some efforts have been made, e.g., Cheng et al. tried the way of using porous anodized aluminum oxide as templates [32], Chen et al. tried the high-temperature chemical vapor deposition approach [2], and Ding et al. tried the top-down etching method [33], conclusions can be made that a high-quality *β*-Ga_2_O_3_ nanotube is still difficult to obtain, which severely hinders the development of *β*-Ga_2_O_3_ nanotube-based devices and applications. Upon this issue, we here report a new facial method to fabricate *β*-Ga_2_O_3_ nanotubes using pre-synthesized GaSb nanowires as sacrificial templates. Two-step thermal treatments are exerted on the GaSb nanowires to control the oxidation process and the transformation to tubular structures. The nanotube length, diameter, and shell thickness can be easily tuned with appropriate precursor template nanowires and thermal treating parameters. Moreover, the atomistic structures and optoelectronic properties of the achieved *β*-Ga_2_O_3_ nanotubes are characterized and tested.

## 2. Materials and Methods

### 2.1. Fabrication of β-Ga_2_O_3_ Nanotubes

The preparation of GaSb nanowires was reported elsewhere [34]. First, the GaSb nanowires were put into a quartz-tube furnace and then heated to 500–800 °C with a ramp rate of 4 °C/min in air conditions for 2–8 h. In this stage, the GaSb nanowires are partially oxidized into core–shell structures. Multiple GaSb segments may remain at this stage serving as the core parts. After heating, the furnace was cooled down to room temperature, and the samples were transferred to a vacuum quartz-tube furnace for the second-step heating treatment. The temperatures were set to 600–950 °C with a ramp rate of 10 °C/min for 3 h. In this stage, the inner GaSb cores were mostly removed via decomposition/sublimation, while the outer shells were transformed into *β*-Ga_2_O_3_ nanotubes. Since *β*-Ga_2_O_3_ is stable at this temperature, these treatments thereby lead to tubular structures rather than porous structures.

### 2.2. Characterizations and In Situ TEM Heating Experiments

The morphology, crystalline structure, and composition of the GaSb nanowires and the Ga_2_O_3_ nanotubes were characterized using the FEI Titan 80-300 TEM (ThermoFisher Scientific, Waltham, MA, USA) and the Talos F200X TEM (ThermoFisher Scientific, Waltham, MA, USA). For SEM characterization, the images were collected using the Helios 5 CX DualBeam SEM (ThermoFisher Scientific, Waltham, MA, USA) and the FEI Quanta 200 SEM (ThermoFisher Scientific, Waltham, MA, USA). Raman spectra of the GaSb nanowires and the Ga_2_O_3_ nanotubes were collected using the RAM-PRO-785E spectrometer (Agiltron, Woburn, MA, USA) with a 785 nm laser. XPS data were collected using the ESCA-3400 spectrometer (Shimadzu, Kyoto, Japan). The in situ heating experiments were performed using the Fusion 350ST heating holder combined with the micro-fabricated chips from Protochips Company, Morrisville, NC, USA. In a typical heating experiment, the GaSb nanowires were heated to a target temperature (e.g., 450 °C) with a ramp rate of 5 °C/s. Then, the temperature was increased step-by-step with a step size of 1 °C until sublimation occurred. The structure evolution was monitored via TEM or STEM mode in real-time.

### 2.3. Photoelectric Tests of the β-Ga_2_O_3_ Nanotubes

I–V curves were measured by sweeping the voltage from −5 V to 5 V using Keithley 4200A combined with a probe station. Lamps with 254 nm or near-infrared wide-spectrum source (waveband of 0.75~5 μm with a band peak at around 4 μm) were used as illumination. In the I–V tests of multiple *β*-Ga_2_O_3_ nanotubes, the samples were dispersed in ethyl alcohol and dripped on a chip with interdigital electrodes. In the I–V tests of a single *β*-Ga_2_O_3_ nanotube, an individual nanotube was marked and two square tungsten microelectrodes (side-length of 50 μm and thickness of 150 nm) were fabricated nearby using focused ion beam deposition. Then, tungsten microwires (width of 1.5 μm and thickness of 150 nm) were fabricated to connect the nanotube to the electrodes. Between the two electrodes, an isolation groove (2 μm in width and 5 μm in depth) was sculpted by the focused ion beam to avoid a potential short circuit.

## 3. Results and Discussion

**Figure 1:**
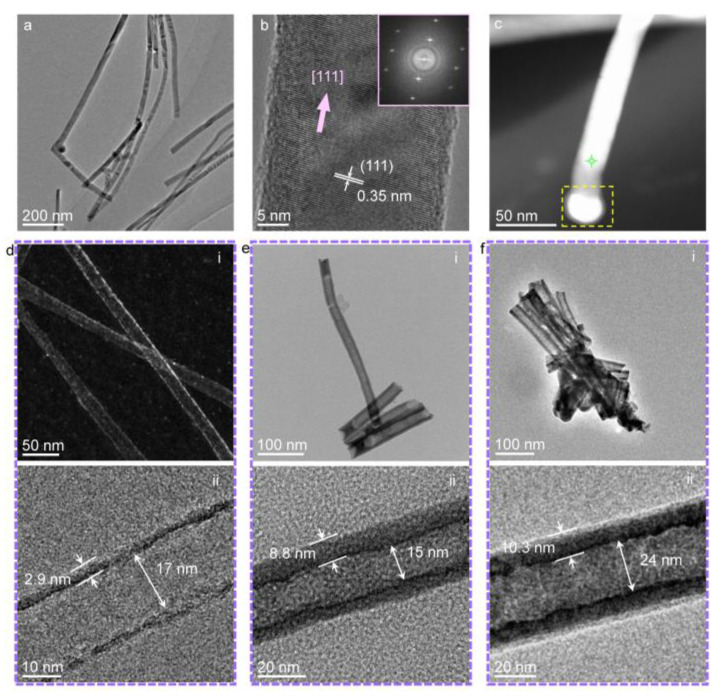
Characterizations of the template GaSb nanowires and the obtained *β*-Ga_2_O_3_ nanotubes. (**a**) TEM image showing the morphology of the pre-synthesized GaSb nanowires. (**b**) High-resolution TEM image of a typical individual GaSb nanowire. The inset shows the fast Fourier transform pattern of the crystalline structure. (**c**) STEM image of a typical GaSb nanowire. The EDS data are acquired at the cross point marked in green and shown in Figure S1 in the Supplementary Materials. The yellow dashed box indicates the residual Au catalysts. (**d**–**f**) STEM and TEM images of the morphologies of the obtained *β*-Ga_2_O_3_ nanotubes after two-step thermal treatments.

Figure 1a shows the transmission electron microscopy (TEM) image of the template GaSb nanowires. As can be seen, the GaSb nanowires have relatively uniform diameters. The high-resolution TEM image in Figure 1b shows a typical individual GaSb nanowire with a diameter of 26.7 nm and a measurable inter-planar spacing of 0.35 nm, corresponding to the (111) crystalline planes [34]. Most of the GaSb nanowires have monocrystalline structures and a preferential growth orientation along [111]. Figure 1c shows the dark-field scanning-TEM (STEM) image of a GaSb nanowire acquired using a high-angle annular detector. The energy dispersive spectrum (EDS) of the nanowire at the point position marked by the green cross (Figure S1) shows that the elemental fraction ratio of Ga to Sb is roughly around 1:1, corroborating the result obtained by the high-resolution TEM images. In some cases, nanoparticles may be retained at the ends of some GaSb nanowires (e.g., the yellow dashed box in Figure 1c). These nanoparticles are Au catalysts (Figure S2) used for the growth of GaSb nanowires and will not influence the further evolution of the GaSb nanowires to *β*-Ga_2_O_3_ nanotubes. Figure 1d–f show the typical products obtained after thermally treating the GaSb nanowires. The solid nanowires are all turned into tubular structures with different thicknesses from 2.9 nm to 10.3 nm relating to different oxidation times. The oxidation times for the nanotubes in Figure 1d, 1e and 1f are 0.1 h, 2 h, and 4 h at 500 °C, respectively.

**Figure 2:**
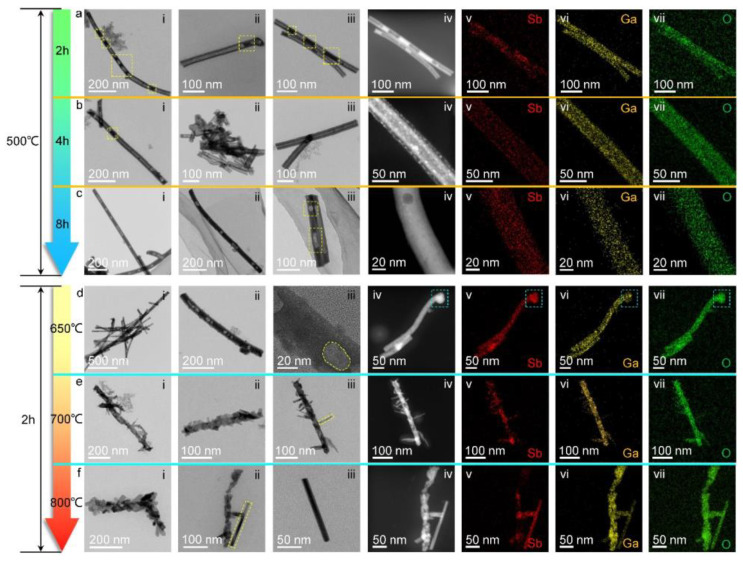
Characterizations of the products obtained in different thermal treating conditions. (**a**–**c**) Morphologies and elemental distributions of the nanostructures which are obtained by heating the template GaSb nanowires in air at 500 °C for 2 h, 4 h, and 8 h, respectively. (**d**–**f**) Morphologies and elemental distributions of the nanostructures which are obtained by heating the template GaSb nanowires in air for 2 h at 650 °C, 700 °C, and 800 °C, respectively.

The critical points of the two-step thermal treatments are the precise control of surface oxidation and the subsequent removal of the residual core parts. As the determinant of controlling the shell thickness, the first-step thermal treatments are carefully examined and shown in Figure 2. Figure 2a(i–iii) shows the morphologies of the products after heating at 500 °C for 2 h in atmospheric conditions. As can be seen, even in this stage, the incipient tubular structures can be identified. However, multi-segments are still encapsulated inside the tubular structures, as marked by the yellow dotted boxes. The STEM image and EDS mappings in Figure 2a(iv–vii) demonstrate that the tubular outer shell (the gray part) mainly consists of Ga and O elements, while the inner multi-segments (the bright parts) are mainly Ga and Sb elements. It indicates that in this thermal treatment stage, tubular structures should be formed via the Kirkendall oxidation process [35,36,37]. According to the literature [38], pristine GaSb nanowires are frequently covered by ultrathin oxide shells (generally smaller than 4 nm at room temperature in air conditions). The oxide shells consist of both Ga_2_O_3_ and Sb_2_O_3_ and prevent the inner GaSb core from further oxidation. In the experiments here, the oxidation of the GaSb nanowires is also very slow when the heating temperature is lower than 200 °C (Figure S3). Thus, raising the heating temperature to enable the secondary oxidation of Sb_2_O_3_ to Sb_2_O_4_ [39,40] allows for the inward diffusion of oxygen and outward diffusion of GaSb known as the Kirkendall effect. Eventually, the Ga_2_O_3_ shells continuously grow at the expense of depleting the inner GaSb.

Figure 2b(i–iii) shows the result of extending the heating time to 4 h at 500 °C. A longer heating time tends to generate thicker oxidation shells together with fewer inner residual GaSb segments. Aside from the increased shell thickness, it is interesting to find that small nanoparticles will form on both the inner and the outer surfaces of the shell during TEM imaging (Figure S4, Video S1). Figure 2b(iv) shows a typical STEM image comprising many nanoparticles represented as bright dots. The EDS mappings shown in Figure 2b(v–vii) indicate that these nanoparticles are Sb-rich, implying that the reaction of 2GaSb + Sb_2_O_3_ → Ga_2_O_3_ + 4Sb has occurred under the energetic irradiation of high-energy electrons [38]. Furthermore, the existence of SbO_x_ in the shell suggests that 4 h thermal heating at 500 °C is insufficient to obtain pure Ga_2_O_3_. Nevertheless, compared with the result of 2 h heating, the formed nanotubes have more porous structures, which can serve as the diffusion channels facilitating further oxidation. Figure 2c(i–iii) shows the morphologies of the products with a much longer heating time of 8 h. The STEM image and EDS mappings shown in Figure 2c(iv–vii) further prove their porous structures and consistent element distributions.

To probe into the feasibility of removing SbO_x_ at elevated temperatures, thermal treatments at 650–800 °C have also been verified. Figure 2d(i–iii) shows the results after thermal treatment at 650 °C for 2 h. Porous structures are observed with more holes inside the nanowires. In some nanowires, SbO_x_ nanoparticles can still be found at the nanowire ends, as indicated by the cyan dashed boxes in Figure 2d(iv–vii). Figure 2e(i–iii) shows the case of thermal treatment at 700 °C for 2 h. The nanowires transformed into a “stem-thorn” configuration with a broken tubular structure as “stem” and nanorods as “thorns”. The STEM image and EDS mappings in Figure 2e(iv–vii) prove that the “thorns” are mainly GaSb_x_O_y_ and possibly formed by the activated regrowth at this high temperature (Figure S5). As for the case at 800 °C, the results are similar to those at 700 °C, as shown in Figure 2f(i–vii). 

**Figure 3:**
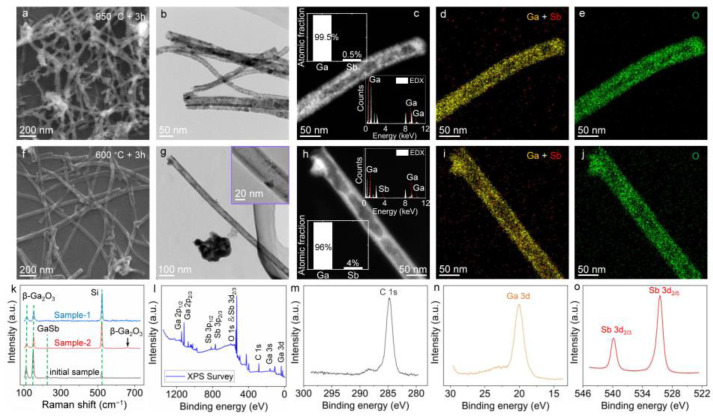
Characterizations of the obtained nanostructures after second-step thermal treatments. (**a**,**b**) SEM and TEM images showing the morphology of Sample-1. (**c**–**e**) STEM image and EDS mappings of a typical nanotube in Sample-1. The inset shows the EDS results in atom fraction. (**f**,**g**) SEM and TEM images showing the morphology of Sample-2. The inset shows the magnified image. (**h**–**j**) STEM image and EDS mappings of a typical nanotube in Sample-2. The inset shows the EDS results in atom fraction. (**k**) Raman spectra of the initial sample, Sample-1, and Sample-2, respectively. (**l**–**o**) XPS measurements of Sample-2.

Apparently, long-time heating and higher temperatures in air conditions are no longer beneficial for the formation of tubular structures as the GaSb_x_O_y_ is hard to remove or convert into tubular Ga_2_O_3_ (Figure S6). This implies that after certain oxidization of the template GaSb nanowires, the sculpting of the residual core parts requires delicate treatments. To achieve this, the second-step thermal heating is performed in vacuum conditions to avoid further reaction and regrowth. Herein, two conditions are verified on the template GaSb nanowires, which have already been heated at 500 °C for 2 h in air conditions. One sample is further heated in vacuum conditions at 950 °C for 3 h (referred to as Sample-1 hereafter), while the other sample is treated at 600 °C for 3 h in vacuum (referred to as Sample-2 hereafter). Figure 3a,b show scanning electron microscope (SEM) and TEM images of the morphology of Sample-1. As can be seen, the residual GaSb cores after the first-step thermal treatment have been successfully removed with nanotubes left. Unfortunately, the nanotubes are quite porous (Figure 3b,c), possibly due to the excessive heating temperature in vacuum conditions. The EDS result shown in the inset of Figure 3c shows that most of the Sb element disappears (Figure S7). The EDS mappings in Figure 3d,e suggest that the resulting nanotubes are mainly Ga_2_O_3_. For Sample-2, as shown in Figure 3f,g, the obtained nanotubes have much more even structures, indicating that a temperature of 600 °C is safe to preserve the Ga_2_O_3_ shell and sufficient to remove the GaSb cores. Thereby, nearly complete Ga_2_O_3_ nanotubes can be successfully achieved. The STEM image and EDS data in Figure 3h–j indicate that the residual Sb element is less than 4% in the atom fraction (Figure S7) and the tubular structures corroborate Ga_2_O_3_. 

To reveal the crystal type of the products after thermal treatments, Raman measurements are performed and shown in Figure 3k. Raman spectra are acquired on each sample, i.e., the initial sample which stands for the template GaSb nanowires after the first thermal treatment at 500 °C for 2 h in air conditions (black line), Sample-1 which undergoes a second thermal treatment at 950 °C for 3 h in vacuum (blue line), and Sample-2 which undergoes a second thermal treatment at 600 °C for 3 h in vacuum (red line). It can be seen that the GaSb peak at 226 cm^−1^ of the initial sample is clear due to the existence of GaSb cores, while this peak almost disappears in Sample-1 and Sample-2 as GaSb decomposes and sublimates in vacuum conditions (Figure S8) [41]. The featured Raman peaks at 111 cm^−1^, 150 cm^−1^, and 660 cm^−1^ of Sample-1 and Sample-2 indicate that the obtained nanotubes are all *β*-Ga_2_O_3_ [42]. Moreover, Figure 3l–o show the X-ray photoelectron spectroscopy (XPS) measurements of Sample-2. The XPS result in Figure 3l confirms the existence of Ga, Sb, and O elements. The characteristic binding energy peak of Ga 3d at ~20.3 eV in Figure 3n is ascribed to *β*-Ga_2_O_3_ [43,44]. The binding energy peaks of Sb 3d_2/3_ and Sb 3d_2/5_ at 539.9 eV and 530.5 eV in Figure 3o correspond to that of Sb_2_O_4_ very well [45]. This evidence confirms that the structures of Sample-2 are *β*-Ga_2_O_3_ nanotubes.

**Figure 4:**
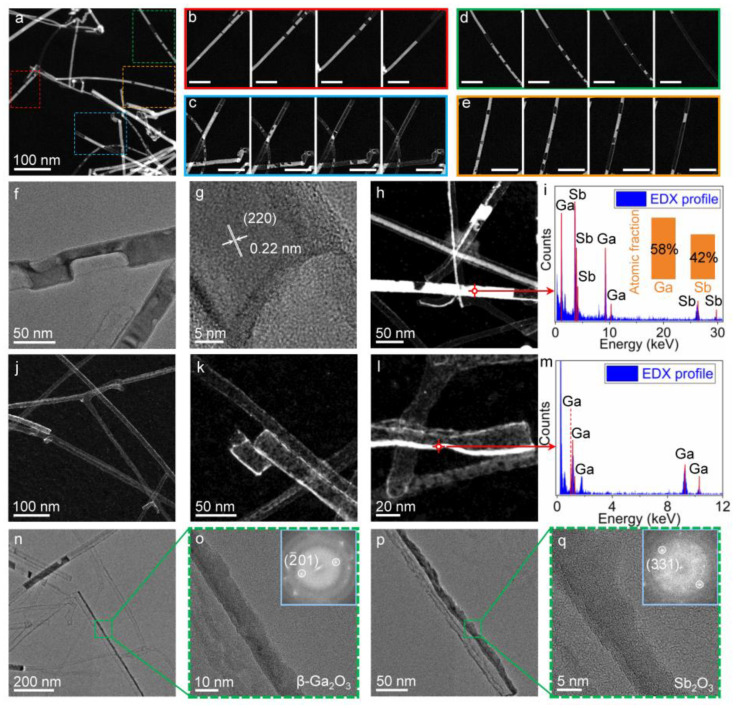
Revealing the dynamic process of nanotube formation in situ. (**a**) STEM image of template GaSb nanowires heated at 500 °C. (**b**–**e**) STEM image sequences of GaSb nanowires during sublimation. All scale bars are 50 nm. (**f**,**g**) High-resolution TEM images of the atomistic structures of GaSb nanowires during sublimation. (**h**,**i**) STEM image and EDS result of a GaSb nanowire during sublimation. (**j**,**k**) STEM images of the obtained nanotubes after sublimation. (**l**,**m**) STEM image and EDS result of an obtained nanotube in its final state. (**n**,**o**) TEM images showing the obtained *β*-Ga_2_O_3_ nanotubes. (**p**,**q**) TEM images showing the residual Sb_2_O_3_ nanostructures.

The second-step heating treatment in vacuum conditions well avoids the complex reactions observed in air conditions during the first-step heating treatment. This implies that the residual core parts after the first-step thermal treatment should undergo a physical evolution like sublimation or evaporation. To understand this process and the mechanism lying behind it, in situ TEM heating experiments are performed to reveal the dynamic evolution process. Figure 4a shows a dark-field image of in situ heating of the GaSb nanowires, which have been exposed to air for oxidation for a while in advance. When the heating temperature is increased to around 500 °C, sublimation of the GaSb nanowires can be observed (Video S2). Figure 4b–e show some typical image sequences of the sublimation process. The continuous decrease in the GaSb segments indicates that all the unoxidized GaSb parts can be completely removed. Figure 4f,g show the atomistic structures of two GaSb nanowires during sublimation. It can be seen that the GaSb nanowires maintain their crystalline structures, but lose substances like sublimating/evaporating liquids. Figure 4h,i give a STEM image and the EDS result of the GaSb nanowires when sublimation is halted. The Ga and Sb elements are found to be Ga:Sb = 1.38:1 in the atom fraction. The slight deviation of the ratio from 1:1 may possibly be caused by the preferential sublimation of Sb, which has a higher vapor pressure than Ga under this temperature [46]. Thereby, GaSb may vanish via both decomposition and sublimation. Figure 4j–l show the achieved nanotubes when all the GaSb parts are depleted. It can be seen that the slight pre-oxidation in air only yields very thin nanotubes. The EDS result in Figure 4m shows that Sb can hardly be found, differing from the results in Figure 3o where residual Sb still can be observed. It should be pointed out that the removal of Sb elements relies on the formed oxide shells to some extent. Generally, the Sb element is hard to be completely removed once thick oxide shells are formed. This may be due to the confinement of the oxide shells which inhibit further sublimation. In the cases where only thin oxide shells are formed, the inner GaSb cores are easily removed to form pure thin *β*-Ga_2_O_3_ nanotubes/nanowires. The high-resolution TEM images in Figure 4n,o, as well as the fast Fourier transform pattern, reveal a featured crystalline plane corresponding to the ($\overset{–}{2}$01) of *β*-Ga_2_O_3_. This largely interprets the formation mechanism of *β*-Ga_2_O_3_ nanotubes in the aforementioned second-step thermal treatments. Noticeably, in a few nanowires where antimony oxide is formed, it is hard to remove it with in situ thermal heating. One typical residual antimony oxide is shown in Figure 4p,q. The crystalline plane is found to correspond to the (301) of Sb_2_O_3_. Nevertheless, this issue can be solved by properly increasing the heating temperature.

**Figure 5:**
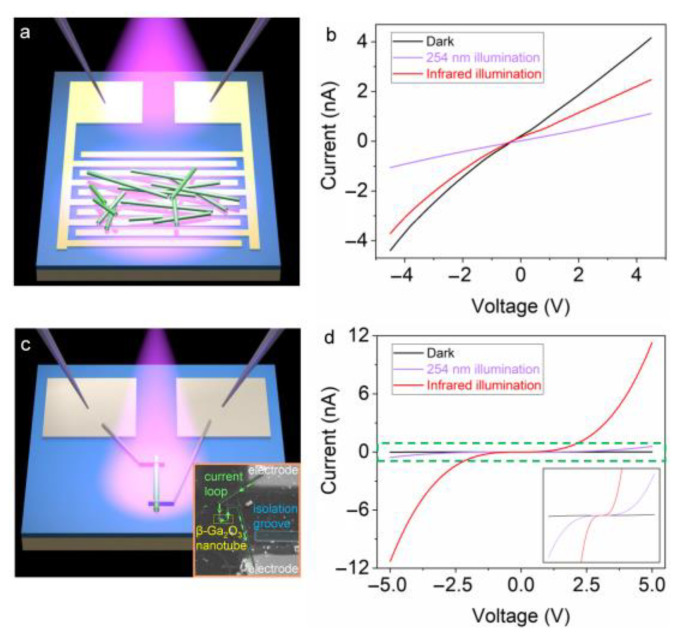
Photoelectric test of the obtained *β*-Ga_2_O_3_ nanotubes. (**a**) Schematic illustration of the I–V test of multiple *β*-Ga_2_O_3_ nanotubes using a chip with interdigital micro-electrodes. (**b**) I–V curves of multiple *β*-Ga_2_O_3_ nanotubes measured in dark condition (black line), under 254 nm illumination (purple line), and under near-infrared illumination (red line), respectively. (**c**) Schematic illustration of the setup for measuring a single *β*-Ga_2_O_3_ nanotube. The inset shows an SEM image of the experiment. (**d**) I–V curves of the single *β*-Ga_2_O_3_ nanotube measured in dark condition (black line), under 254 nm illumination (purple line), and under near-infrared illumination (red line), respectively. The inset shows the magnified region marked by the green dashed box.

To uncover the photoelectric properties of the obtained *β*-Ga_2_O_3_ nanotubes, micro-electrodes are fabricated to perform I–V tests under illumination on the nanotubes. Figure 5a shows the illustration of the setup of the photoelectric test. The I–V curves in dark condition (black line), under 254 nm illumination (purple line), and under near-infrared illumination (red line) are plotted together in Figure 5b. It can be seen that the dark current is higher than the photocurrents generated by either 254 nm or near-infrared illumination. The randomly distributed composite structures possibly comprise both *β*-Ga_2_O_3_ nanotubes and residual GaSb. This makes the photoelectric test complicated and hard to interpret. Moreover, the negative photoconductivity effect may also play a critical role [47,48]. In order to suppress such interference, a single *β*-Ga_2_O_3_ nanotube was constructed to perform a test, as shown in Figure 5c. The inset shows an SEM image of the as-fabricated configuration of the electrodes and the *β*-Ga_2_O_3_ nanotube (yellow dotted box) (Figure S9). Similar I–V tests in dark conditions (black line), under 254 nm illumination (purple line), and under near-infrared illumination (red line) are carried out and plotted in Figure 5d. It can be seen that the measured photocurrent at a voltage of 5 V is 14 pA in the dark condition, while this value increases to 577 pA in the case of 254 nm illumination. Generally, the photoresponsivity *R_λ_* is given by

(1) $$R_{\lambda }=\frac{I_{photo}-I_{dark}}{P_{\lambda }S}$$

where the *I_photo_* and *I_dark_* are the photocurrent and dark current, respectively, *P_λ_* is the power density of the light, and *S* is the effective illuminated area. In our experiments, the diameter and length of the single *β*-Ga_2_O_3_ nanotube are 35 nm and 3.8 μm, respectively. The effective illuminated area is calculated to be ~2.09 × 10^−9^ cm^2^. *P_λ_* is 10.4 mW/cm^2^. Therefore, the photoresponsivity *R_λ_* is calculated to be ~25.9 A/W. In addition, the detectivity D* can be obtained through the equation

(2) $$D^{*}=\frac{R_{\lambda }}{\sqrt{2e\times I_{dark}/S}}$$

where *e* is the electron charge (1.6 × 10^−19^ C). In our case, D* is calculated to be ~5.6 × 10^11^ Jones. These results indicate that the obtained nanotubes possess striking photoelectric properties. Moreover, when the nanotube is illuminated by a near-infrared light source, the photocurrent is measured to be 11.3 nA at 5 V, showing a response ratio (*I_photo_*/*I_dark_*) as high as 807. As the dark current in Figure 5d (black line in the inset) is relatively low, the noise caused by the dark current of this device is thus not discussed here. Furthermore, the leakage current of the device is measured to be at the level of 5 pA under a voltage of 5 V (Figure S10). It is a rather small value and thereby ignored. The high photo-responses at both the ultraviolet band and the near-infrared band imply a complex result of the nanotube. One possible reason is that some tiny GaSb segments may still be retained inside the *β*-Ga_2_O_3_ nanotube but can be hardly seen using SEM characterization. Hence, the nanotube shows unexpected responses under the excitation of a near-infrared light source. Further optimization of the preparation method is still required to achieve pure *β*-Ga_2_O_3_ nanotubes. Also, the intrinsic photoelectric property of *β*-Ga_2_O_3_ nanotubes should be further investigated with GaSb influences completely excluded.

## 4. Conclusions

In summary, *β*-Ga_2_O_3_ nanotubes are successfully prepared using a simple two-step heating method using GaSb nanowires as sacrificial templates. The characteristics of the *β*-Ga_2_O_3_ nanotubes including length, diameter, and shell thickness can be tuned easily by regulating the GaSb-nanowires templates and the heating parameters. Kirkendall oxidation and sublimation are revealed to be critical determinants for the formation of *β*-Ga_2_O_3_ nanotubes. And this mechanism is further evidenced via in situ TEM heating experiments. Moreover, the photoelectric properties of the obtained *β*-Ga_2_O_3_ nanotubes have been investigated. With a 254 nm ultraviolet illumination, a photoresponsivity of ~25.9 A/W and a detectivity of ~5.6 × 10^11^ Jones are achieved on a single-*β*-Ga_2_O_3_ nanotube device.

## Data Availability

All the data supporting the findings of this study are available within this article and its Supplementary Materials files. All raw images and source data are available from the authors upon reasonable request.

## Supplementary Materials

The following supporting information can be downloaded at: https://www.mdpi.com/article/10.3390/nano13202756/s1, Figure S1: EDS results of the point marked in Figure 1c; Figure S2: Characterizations of the Au tips in some GaSb nanowires; Figure S3: Characterization of the products obtained by heating the GaSb nanowires; Figure S4: Nanoparticle formation when the nanotube is exposed to a high-energy electron beam; Figure S5: Characterization of the products obtained by heating GaSb nanowires; Figure S6: Characterization of the products obtained by heating GaSb nanowires; Figure S7: Characterization of the nanotubes in Sample-1 and Sample-2; Figure S8: Raman peaks revealing the disappearance of GaSb. Figure S9: Enlarged SEM image of the device. Figure S10: The leakage current of the device shown in Figure 5c. Video S1: Formation process of Sb-rich nanoparticles when exposed to the high energy electron beam; Video S2: Sublimation process of GaSb nanowires at 500 °C.
