# Supplementary material for: Fabrication of β-Ga2O3 Nanotubes via Sacrificial GaSb-Nanowire Templates
Source: Nanomaterials (Basel). 2023 Oct 13;13(20):2756. doi: 10.3390/nano13202756 (PMC10609696; doi:10.3390/nano13202756)
Supplement: Supplementary file 1 [file nanomaterials-13-02756-s001.zip › nanomaterials-2602393-supplementary.pdf]

Supporting Information

Fabrication of β-Ga_2O_3 Nanotubes via Sacrificial GaSb-Nanowire Templates

Lei Shangguan [1,2,†], Long-Bing He [1,2,*,†], Sheng-Pan Dong [2], Yu-Tian Gao [1], Qian Sun [1], Jiong-Hao Zhu [2], Hua Hong [1], Chao Zhu [1], Zai-Xing Yang [3,4] and Li-Tao Sun [1]

[1] SEU-FEI Nano-Pico Center, Key Laboratory of MEMS of Ministry of Education, Southeast University, Nanjing 210096, China; 230198521@seu.edu.cn (L.S.); 220221733@seu.edu.cn (Y.-T.G.); 220221749@seu.edu.cn (Q.S.); huahong@seu.edu.cn (H.H.); phczhu@seu.edu.cn (C.Z.); slt@seu.edu.cn (L.-T.S.)
[2] SEU-AMTE Collaborative Center for Atomic Layer Deposition and Etching, Southeast University, Wuxi 214000, China; 19906783878@163.com (S.-P.D.); 220226133@seu.edu.cn (J.-H.Z.)
[3] School of Physics, Shandong University, Jinan 250100, China; zaixyang@sdu.edu.cn
[4] School of Microelectronics, Shandong University, Jinan 250100, China
* Correspondence: helongbing@seu.edu.cn
† These authors contributed equally to this work.

Characterization of the GaSb nanowires

Figure S1 shows the EDS results of the point marked in Figure 1c in the manuscript.

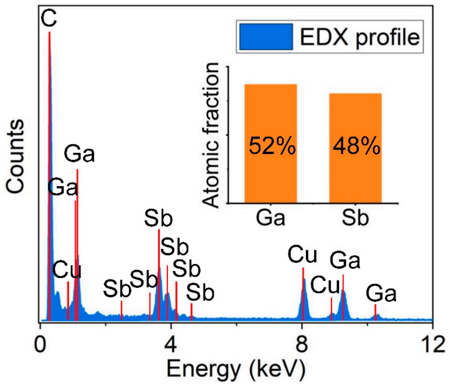

Figure S1. EDS results of the point marked in Figure 1c.

Characterization of the Au tips in some GaSb nanowires

Figure S2a-c show the Au tips of some GaSb nanowires. The Au nanoparticles are catalysts, and some of them break off from the nanowires during sample transfer, as shown in Figure S2d.

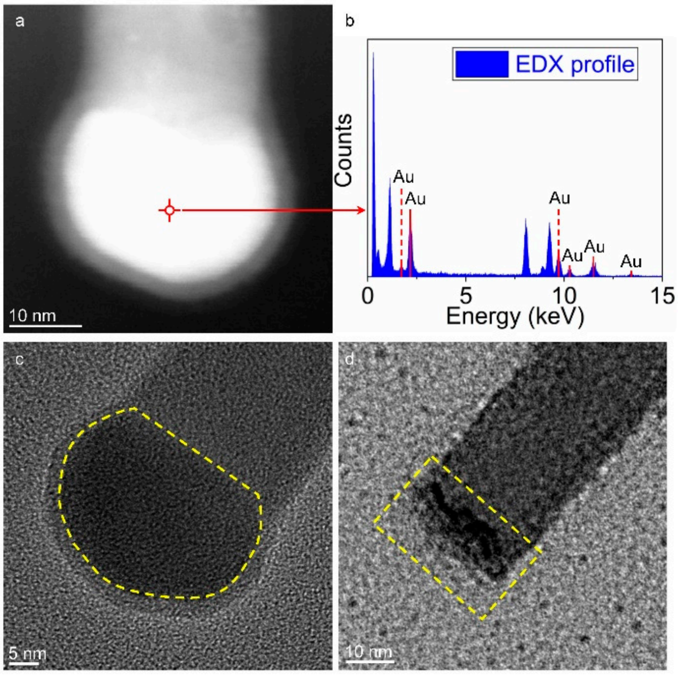

Figure S2. Characterizations of the Au tips in some GaSb nanowires. a) STEM image of the pristine GaSb nanowire with a Au tip. b) EDS results of the point marked in (a). c-d) TEM images of the GaSb nanowires with and without Au tips, respectively.

Heating treatments with different temperatures for 2 h

In addition to the cases shown in Figure 2 in the manuscript, some more heating temperatures (with a fixed heating time of 2 h) have been verified and shown in Figure S3a-o. When the heating temperature is 200 °C, there is no obvious change in the GaSb nanowires (Figure S3a-b). When the heating temperatures are higher than 500 °C, porous nanotubes can be obtained as shown in Figure S3c-e, 3f-j, and 3k-o, respectively. Moreover, Sb-rich "thorns" are found on the samples in Figure S3k-o, similar to the phenomena in Figure 2e-f in the manuscript.

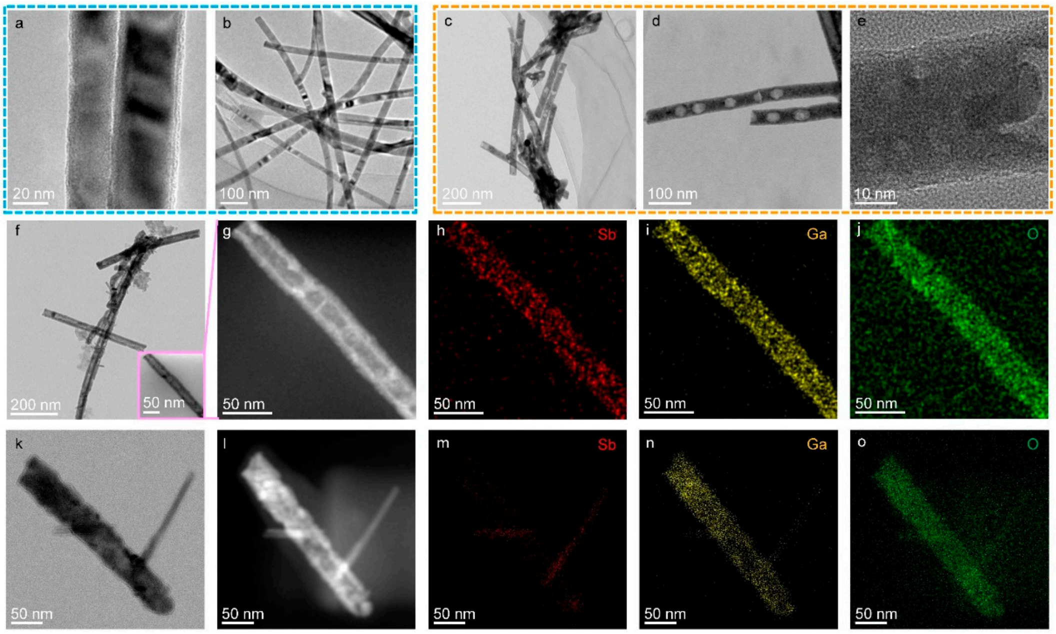

Figure S3. Characterization of the products obtained by heating the GaSb nanowires. a-b) TEM images of the products obtained by heating the GaSb nanowires at 200 °C for 2 h. c-e) TEM images of the products obtained by heating the GaSb nanowires at 600 °C for 2 h. f-j) TEM images and EDS mappings of the products obtained by heating GaSb nanowires at 700 °C for 1 h and 500 °C for 1 h. k-o) TEM images and EDS mappings of the products obtained by heating GaSb nanowires at 750 °C for 1 h and 800 °C for 1 h.

Formation of Sb-rich nanoparticles on the nanotube

Figure S4 shows the formation of Sb-rich nanoparticles when the nanotubes are exposed to the high-energy electron beam. As shown in Figure S4a, the initial inner and outer diameters of this nanotube are measured to be 16 nm and 35.2 nm, respectively. The black arrow in Figure S4b indicates a very small nanoparticle formed on the outer surface at the early stage. Under longer irradiation/imaging, more nanoparticles are observed, as shown in Figure S4c. The nanoparticles grow quickly (Figure S4d-f). In Figure S4f, the nanoparticles are found to have sizes of around 1.5~2 times larger than those in Figure S4c. During this process, the inner diameter of this nanotube almost remains unchanged while the outer diameter increases from 16 nm (Figure S4a) to 42.3 nm (Figure S4f). Figure S4g-j show the STEM image and the EDS mappings of a nanotube with nanoparticles. The EDS indicates that the as-formed nanoparticles are Sb-rich nanoparticles.

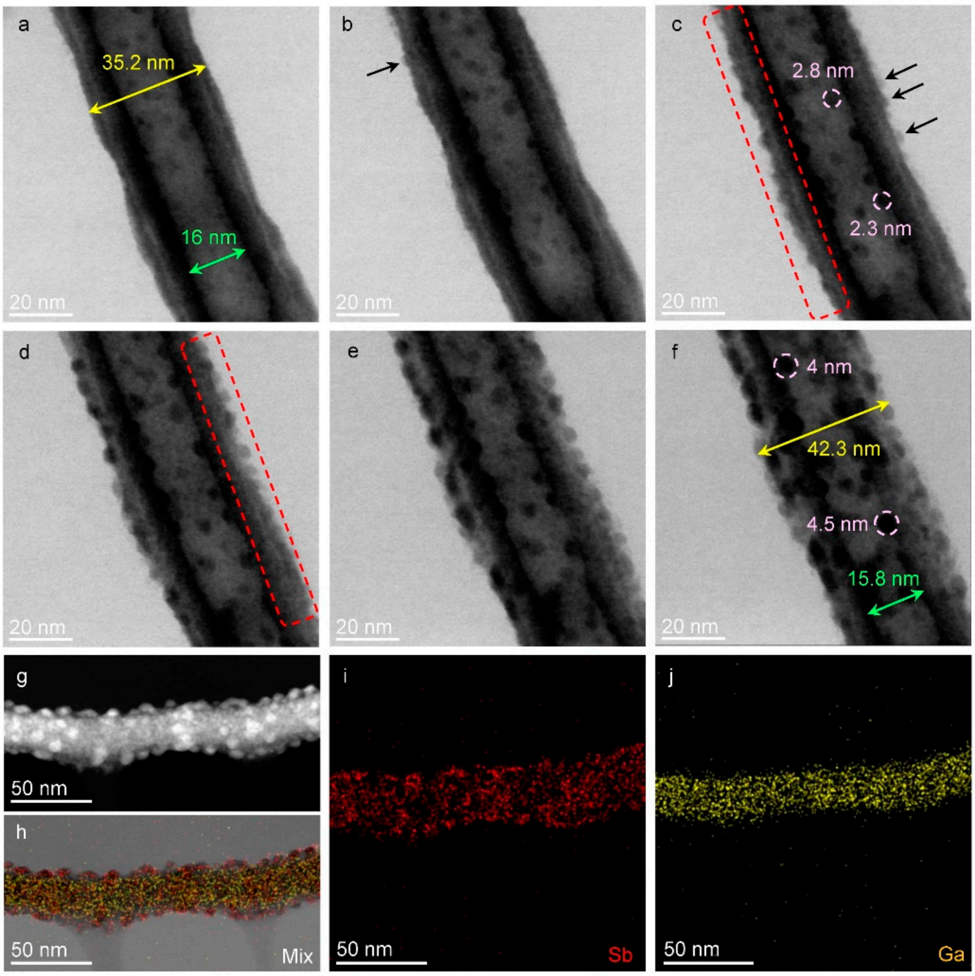

Figure S4. Nanoparticle formation when the nanotube is exposed to a high-energy electron beam. a-f) Sequential TEM snapshots showing the formation of Sb-rich nanoparticles. g-j) STEM image and EDS mappings of a nanotube with as-formed nanoparticles.

Heating treatments using different temperatures and times

Figure S5 shows the products obtained by heating GaSb nanowires at 700 °C for 8 h. The "stem-thorn" structures can be observed, similar to the results in Figure 2e. Noticeably, the Sb-rich "thorns" are denser than that in Figure 2e. It is believed that the number of the formed nano-"thorns" is mainly determined by the heating time.

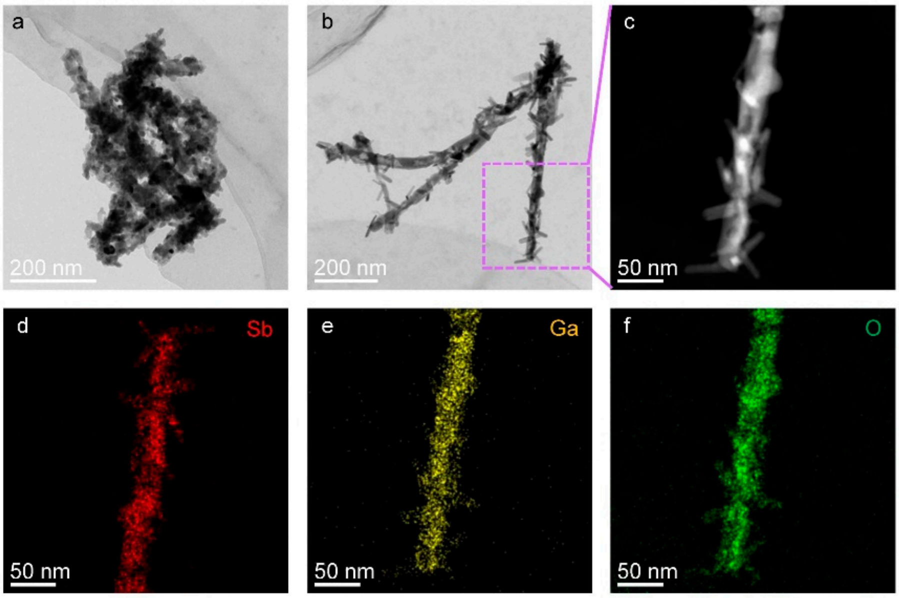

Figure S5. Characterization of the products obtained by heating GaSb nanowires. a-b) TEM images of the products obtained by heating GaSb nanowires at 700 °C for 8 h. c) STEM image of the region marked in purple in (b). d-f) EDS mappings of the nanostructure in (c).

As is discussed in the manuscript, porous structures usually appear when the heating treatment is done over 500 °C for 2 h. To probe into the influence of heating time, the GaSb nanowires are heated over 500 °C but only for 1 h. Figure S6a-e show the case of 600 °C for 1 h. It can be seen that SbO_x still retains and that some small nanoparticles exist inside the nanotube. Figure S6f-j show the case of 700 °C for 1 h. As can be seen, more nanoparticles appear inside the nanotubes. The case of 800 °C for 1 h has also been carried out. A direct comparison between the case of 700 °C (Figure S6k-l) and the case of 800 °C (Figure S6m-n) has been made. It can be found that the small nanoparticles start to diffuse and grow up when the heating temperature is further elevated. They may serve as the seeds for the growth of “thorns” observed in Figure S5 and Figure 2e-f.

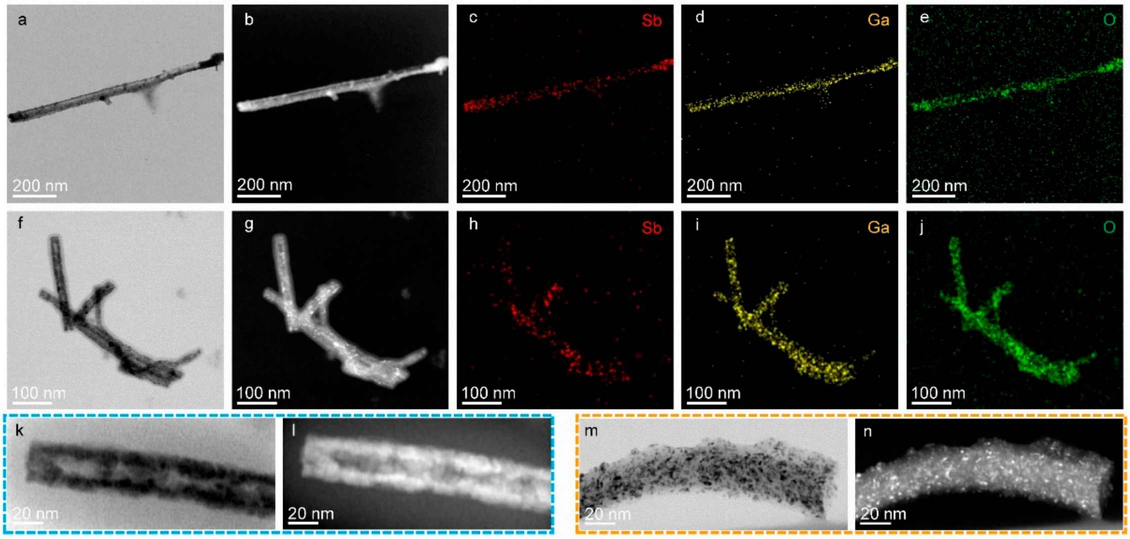

Figure S6. Characterization of the products obtained by heating GaSb nanowires. a-e) The case of heating GaSb nanowires at 600 °C for 1 h. f-j) The case of heating GaSb nanowires at 700 °C for 1 h. A comparison between the typical products in the case of 700 °C for 1 h (k-l) and in the case of 800 °C for 1 h (m-n).

Figure S7a-b show the SEM image and EDS results of Sample-1 while Figure S7c-d show the SEM image and EDS results of Sample-2. The atom fractions of both the two samples derived from the EDS spectra are in good agreement with the results in Figures 4c and 4h in the manuscript. The SEM image in Figure S7c shows the morphology of the as-prepared β-Ga_2O_3 nanotubes (i.e. Sample-1) in a large region.

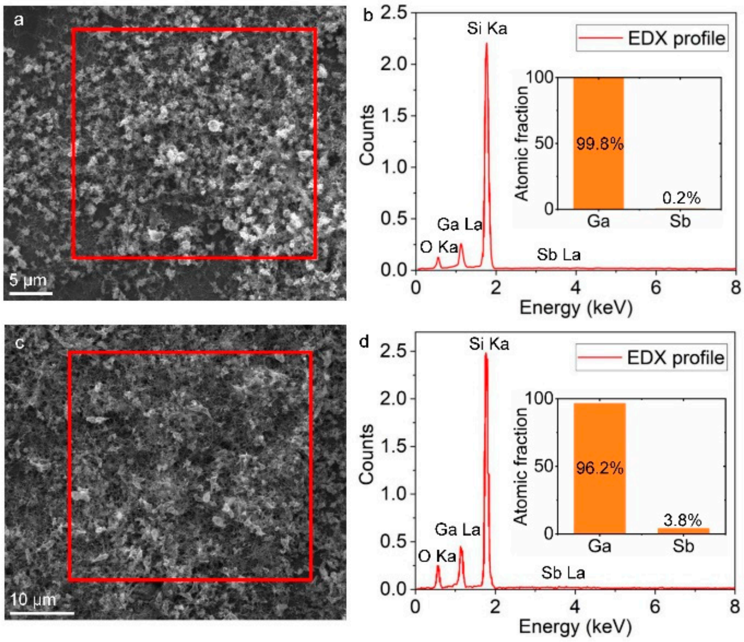

Figure S7. Characterization of the nanotubes in Sample-1 and Sample-2. a) SEM image of Sample-1. b) EDS spectrum of the region marked in red in (a). c) SEM image of Sample-2. d) EDS spectrum of the region marked in red in (c).

Figure S8a shows the Raman spectra of the initial sample, Sample-1, and Sample-2, respectively. The area indicated in yellow is enlarged and shown in Figure S8b. It indicates the disappearance of GaSb signal after the thermal treatment in vacuum.

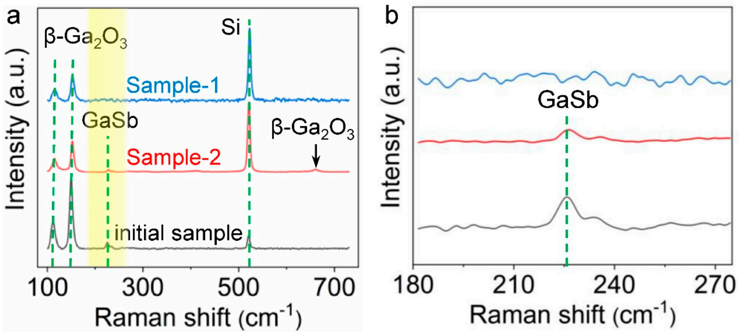

Figure S8. Raman spectra revealing the disappearance of GaSb. a) Raman spectra of the initial sample, Sample-1, and Sample-2, respectively. b) Magnified region of the Raman peaks marked in yellow in (a).

Figure S9 shows the SEM image of the β-Ga_2O_3 nanotube device.

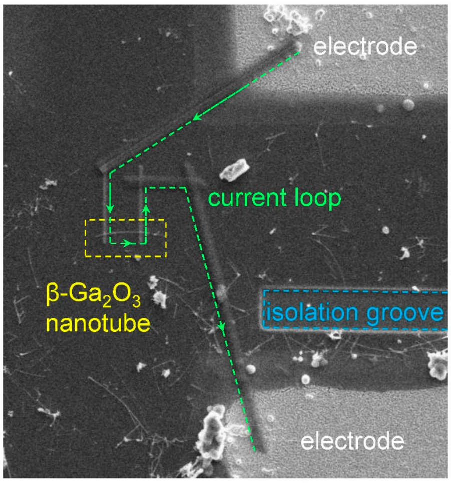

Figure S9. SEM image of an as-fabricated β-Ga_2O_3 nanotube device.

Figure S10 shows the leakage current of device shown in Figure 5c when the voltage varies from -5~5 V.

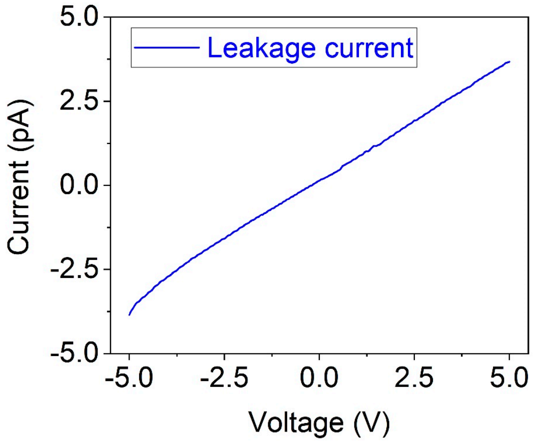

Figure S10. The leakage current of the device shown in Figure 5c.
